# Supplementary material for: The role of parasite-produced dopamine in Toxoplasma gondii-altered host behaviour
Source: Nat Commun. 2025 Dec 5;16:10906. doi: 10.1038/s41467-025-66139-3 (PMC12680685; doi:10.1038/s41467-025-66139-3)
Supplement: Supplementary file 1 — Supplementary Information [file 41467_2025_66139_MOESM1_ESM.pdf]

Supplementary Information

Supplementary Note

Minimum sample size

Minimum sample size was calculated by Mead's Resource Equation E=N-T [1], where E is the error degrees of freedom (in the range of 10-20), N the total degree of freedom (1 less than total number of animals), and T the number of treatments. Thus, since we had 5 lines and 2 sexes T = 5 x 2 = 10, and by taking E = 20, we have that N = E + T = 20 + 10 = 30, resulting in a minimum sample size of (30-1)/10 ≅ 3 animals per group. The sample size used was thus marginally higher than powered to accommodate both for the subtlety of the predicted effects and for potential exclusion of any *T. gondii* exposed but subsequently serologically/cyst-negative individuals from analyses, as had been documented previously for wildtype *T. gondii* [2] and may be predicted here where using novel GM OE *T. gondii* lines for the first time in rat hosts.

Table S1. Number of rats examined per round by experimental line.

Line	Round 1	Round 2	Round 3
Control	12	6	6
WT	12		11
*Tg*TH0		12	
*Tg*TH-MID		11	
*Tg*TH-HIGH			15

Table S2. Number of rats of each sex examined by experimental line.

Line	Male	Female
Control	12	12
WT	11	12
*Tg*TH0	6	6
*Tg*TH-MID	5	6
*Tg*TH-HIGH	7	8

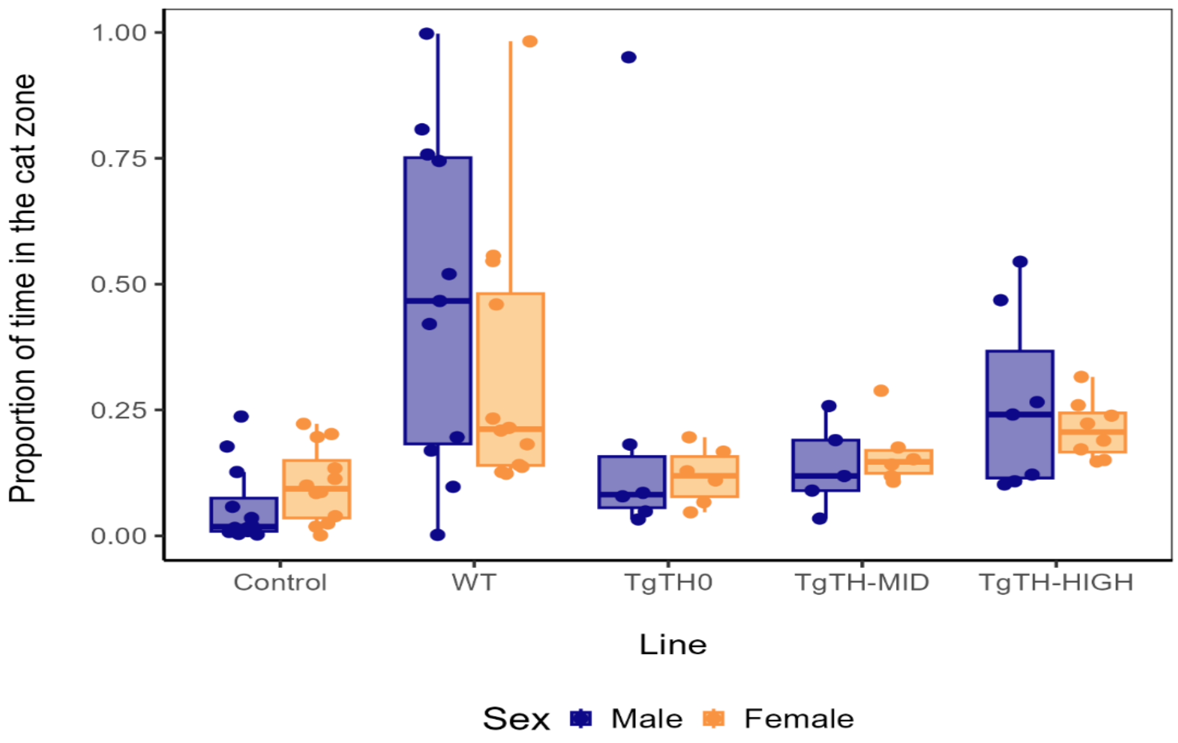

Figure S1. Proportion of time spent in the cat zone. Distribution of the observations by line and sex (males-blue: females-orange). Number of rats per male/female line group: Control = 12/12, WT = 11/12, *Tg*TH0 = 6/6, *Tg*TH-MID = 5/6 and *Tg*TH-HIGH = 7/8. Source data are provided as a Source Data file.

Table S3. Proportion of time spent in the cat zone. Posterior means and 95% credible intervals (CrIs; see Figure 1).

Line	Mean	95% CrI lower limit	95% CrI upper limit
Control	0.084	0.054	0.128
WT	0.443	0.324	0.571
*Tg*TH0	0.107	0.075	0.153
*Tg*TH-MID	0.155	0.111	0.209
*Tg*TH-HIGH	0.243	0.185	0.315

Table S4. Proportion of time spent in the cat zone. Posterior mean differences between lines and 95% credible intervals (CrIs; see Figure 1). Differences whose 95% credible interval does not include the value of zero are considered relevant and are highlighted in grey.

Lines	Mean	95% CrI lower limit	95% CrI upper limit
Control - *Tg*TH0	-0.023	-0.079	0.031
Control - *Tg*TH-MID	-0.071	-0.134	-0.009
Control - *Tg*TH-HIGH	-0.159	-0.238	-0.088
TgTH0 - *Tg*TH-MID	-0.048	-0.113	0.012
TgTH0 - *Tg*TH-HIGH	-0.136	-0.213	-0.065
TgTH-MID - *Tg*TH-HIGH	-0.088	-0.170	-0.010
WT - Control	0.359	0.235	0.489
WT - *Tg*TH0	0.336	0.210	0.464
WT - *Tg*TH-MID	0.288	0.160	0.420
WT - *Tg*TH-HIGH	0.200	0.067	0.339

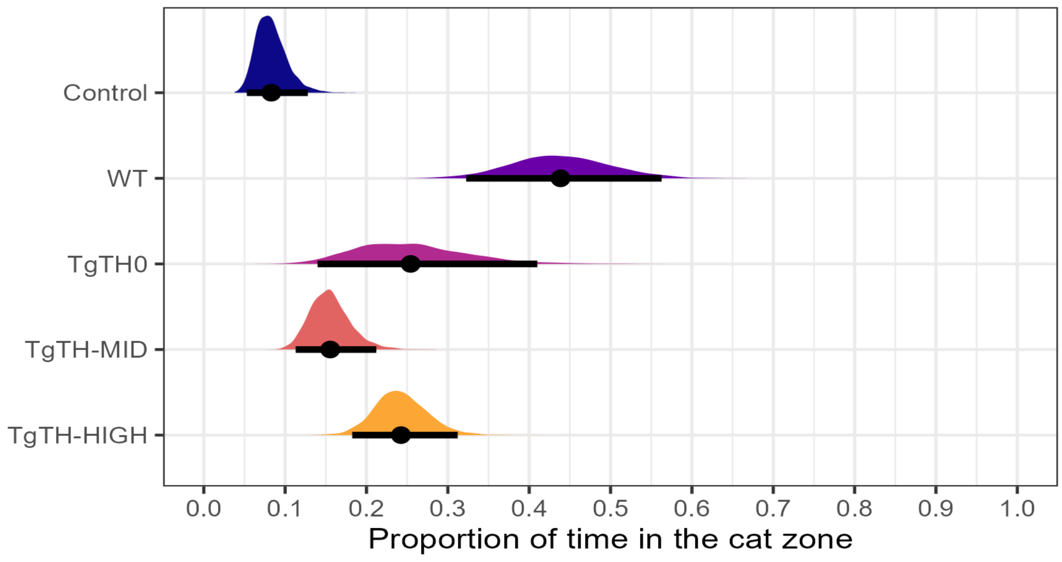

Figure S2. Proportion of time spent in the cat zone (including the extreme value). Posterior distributions of the mean and 95% credible intervals, by experimental line (where: uninfected controls – navy; wildtype – purple; *Tg*TH0 no-overexpression – pink; *Tg*TH-MID middle-level overexpression – red; *Tg*TH-HIGH – high overexpression – orange). Number of rats per line group: Control = 24, WT = 23, TgTH0 = 12, TgTH-MID = 11 and TgTH-HIGH = 15. Source data are provided as a Source Data file.

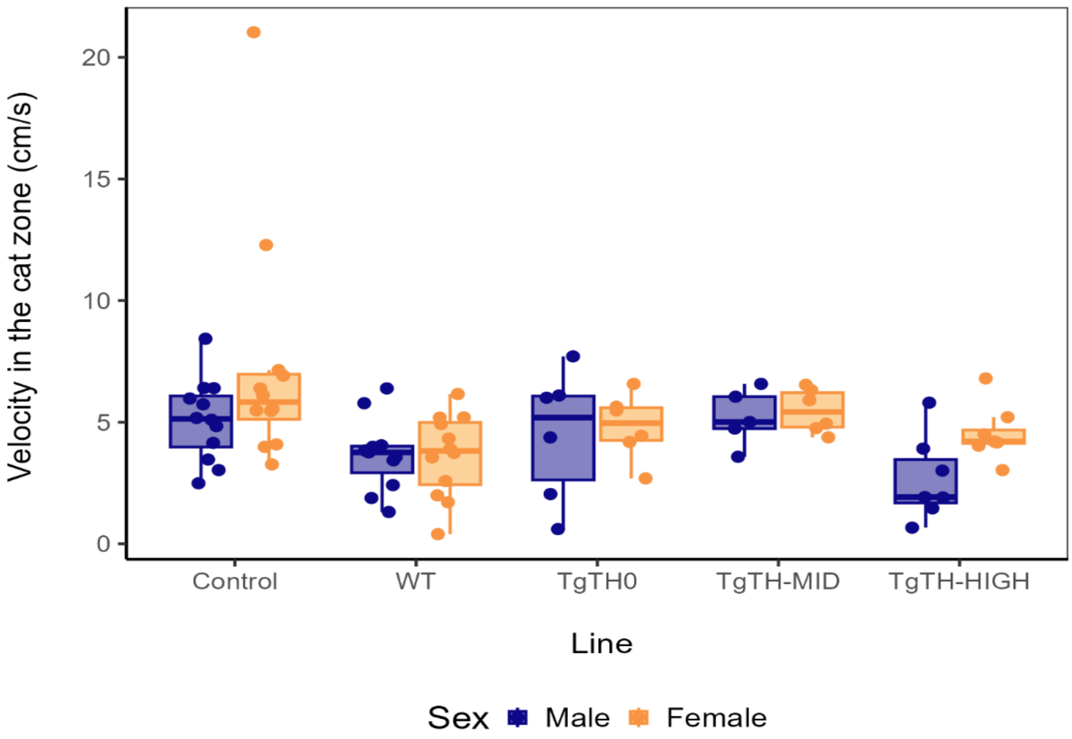

Figure S3. Velocity (cm/s) in the cat zone. Distribution of the observations by line and sex (males blue: females orange). Number of rats per male/female line group: Control = 12/12, WT = 11/12, *Tg*TH0 = 6/6, *Tg*TH-MID = 5/6 and *Tg*TH-HIGH = 7/8. Source data are provided as a Source Data file.

Table S5. Velocity (cm/s) in the cat zone. Posterior means and 95% credible intervals (CrIs; see Figure 2).

Line	Mean	95% CrI lower limit	95% CrI upper limit
Control	5.412	4.720	6.092
WT	3.668	2.966	4.377
*Tg*TH0	4.760	3.711	5.786
*Tg*TH-MID	5.343	4.391	6.276
*Tg*TH-HIGH	3.677	2.804	4.507

Table S6. Velocity (cm/s) in the cat zone. Posterior mean differences between lines and 95% credible intervals (CrIs; see Figure 2). Differences whose 95% credible interval does not include the value of zero are considered relevant and are highlighted in grey.

Lines	Mean	95% CrI lower limit	95% CrI upper limit
Control - *Tg*TH0	0.652	-0.583	1.895
Control - *TgTH*-MID	0.069	-1.120	1.256
Control - *Tg*TH-HIGH	1.735	0.663	2.838
TgTH0 - *Tg*TH-MID	-0.583	-1.986	0.842
TgTH0 - *Tg*TH-HIGH	1.083	-0.212	2.467
TgTH-MID - *Tg*TH-HIGH	1.666	0.359	2.927
WT - Control	-1.744	-2.758	-0.731
WT - *Tg*TH0	-1.092	-2.349	0.162
WT - *Tg*TH-MID	-1.675	-2.823	-0.520
WT - *Tg*TH-HIGH	-0.009	-1.106	1.103

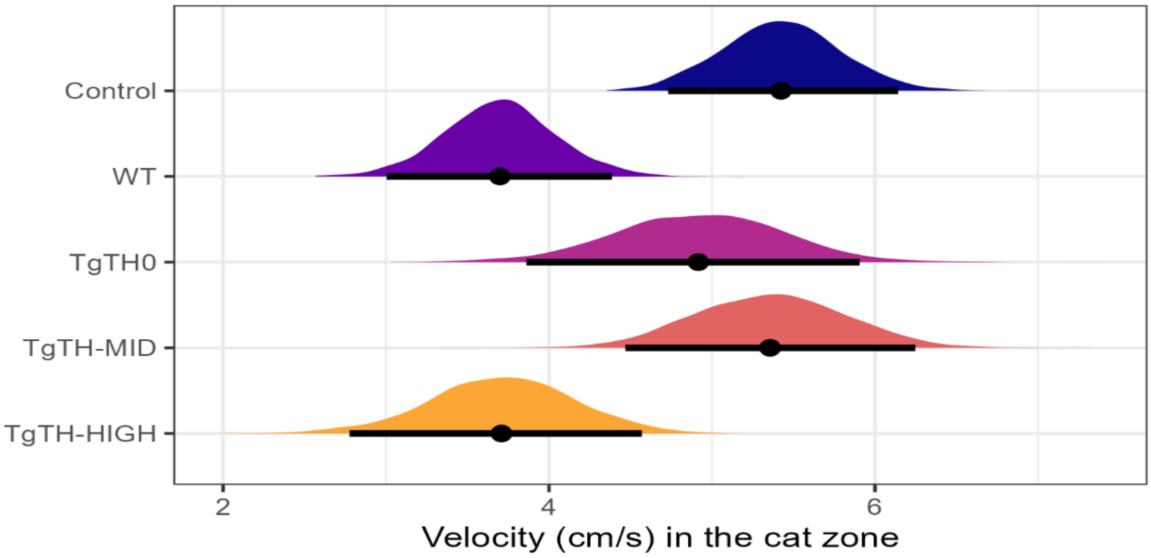

Figure S4. Velocity in the cat zone (including extreme value). Posterior distributions of the mean and 95% credible intervals, by experimental line (where: uninfected controls – navy; wildtype – purple; *Tg*TH0 no-overexpression – pink; *Tg*TH-MID middle-level overexpression – red; *Tg*TH-HIGH – high overexpression – orange). Number of rats per line group: Control = 24, WT = 23, *Tg*TH0 = 12, *Tg*TH-MID = 11 and *Tg*TH-HIGH = 15. Source data are provided as a Source Data file.

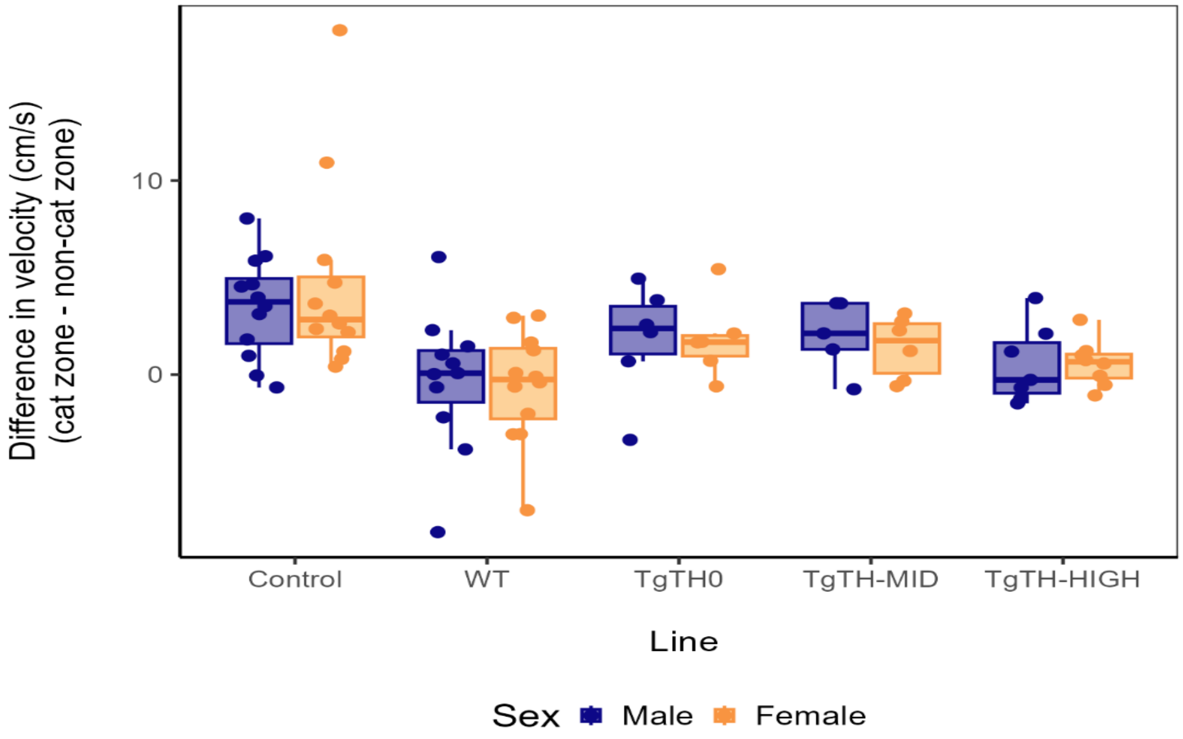

Figure S5. Distribution of observations for difference in velocity (cm/s) between the cat zone and the non-cat zone (cat zone – non-cat zone) by line and sex (where males blue and females orange). Number of rats per male/female line group: Control = 12/12, WT = 11/12, *Tg*TH0 = 6/6, *Tg*TH-MID = 5/6 and *Tg*TH-HIGH = 7/8. Source data are provided as a Source Data file.

Table S7. Difference in velocity (cm/s) in the cat zone and non-cat zone (cat zone – non-cat zone). Posterior means and 95% credible intervals (CrIs; see Figure 3).

Line	Mean	95% CrI lower limit	95% CrI upper limit
Control	3.309	2.247	4.414
WT	-0.159	-1.213	0.862
*Tg*TH0	1.917	0.518	3.327
*Tg*TH-MID	1.749	0.344	3.116
*Tg*TH-HIGH	0.474	-0.636	1.631

Table S8. Difference in velocity (cm/s) in the cat zone and non-cat zone (cat zone – non-cat zone). Posterior mean differences between lines and 95% credible intervals (CrIs; see Figure 3). Differences whose 95% credible interval does not include the value of zero are considered relevant and are highlighted in grey.

Lines	Mean	95% CrI lower limit	95% CrI upper limit
Control - *Tg*TH0	1.393	-0.361	3.192
Control - *Tg*TH-MID	1.561	-0.167	3.339
Control - *Tg*TH-HIGH	2.835	1.252	4.413
TgTH0 - *Tg*TH-MID	0.168	-1.807	2.138
TgTH0 - *Tg*TH-HIGH	1.442	-0.345	3.192
TgTH-MID - *Tg*TH-HIGH	1.274	-0.575	3.047
WT - Control	-3.468	-5.029	-2.003
WT - *Tg*TH0	-2.076	-3.799	-0.365
WT - *Tg*TH-MID	-1.908	-3.653	-0.192
WT - *Tg*TH-HIGH	-0.633	-2.191	0.880

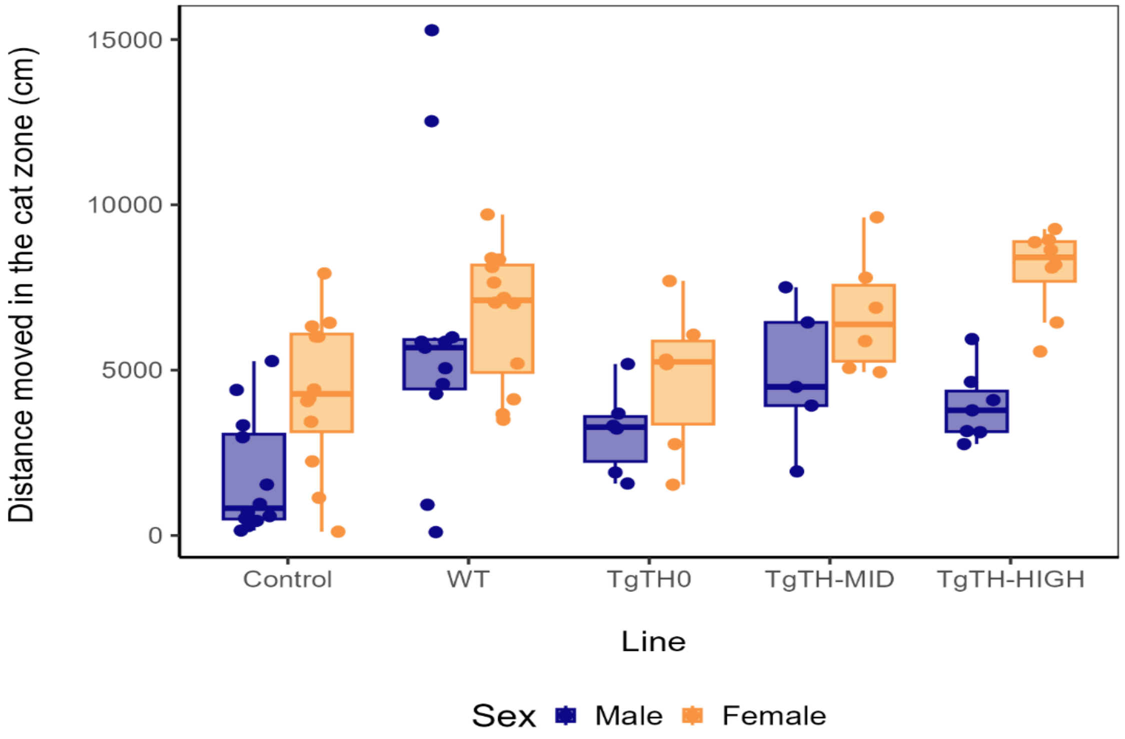

Figure S6. Distance (cm) moved in the cat zone. Distribution of the observations, by line and sex (where males blue and females orange). Number of rats per male/female line group: Control = 12/12, WT = 11/12, *Tg*TH0 = 6/6, *Tg*TH-MID = 5/6 and *Tg*TH-HIGH = 7/8. Source data are provided as a Source Data file.

Table S9. Distance (cm) moved in the cat zone. Posterior means and 95% credible intervals (CrIs; see Figure 4).

Sex	Line	Mean	95% CrI lower limit	95% CrI upper limit
Male	Control	1,796.614	807.409	2,801.757
Female	Control	4,261.909	3,252.688	5,232.110
Male	WT	4,747.165	3,687.648	5,812.523
Female	WT	7,212.460	6,161.477	8,239.566
Male	*Tg*TH0	2,798.066	1,478.664	4,117.265
Female	*Tg*TH0	5,263.361	3,925.289	6,605.784
Male	*Tg*TH-MID	4,457.514	3,101.459	5,797.528
Female	*Tg*TH-MID	6,922.809	5,592.463	8,271.264
Male	*Tg*TH-HIGH	4,789.745	3,635.757	6,000.124
Female	*Tg*TH-HIGH	7,255.040	6,045.978	8,407.114

Table S10. Distance (cm) moved in the cat zone. Posterior mean differences between lines and 95% credible intervals (CrIs; see Figure 4). Differences whose 95% credible interval does not include the value of zero are considered relevant and are highlighted in grey by row.

Sex	Lines	Mean	95% CrI lower limit	95% CrI upper limit
Male	Control - *Tg*TH0	-1,001	-2,544	492
Male	Control - *Tg*TH-MID	-2,661	-4,205	-1,175
Male	Control - *Tg*TH-HIGH	-2,993	-4,339	-1,608
Male	WT - Control	2,951	1,708	4,209
Male	*Tg*TH0 - *Tg*TH-MID	-1,659	-3,435	88
Male	*Tg*TH0 - *Tg*TH-HIGH	-1,992	-3,650	-342
Male	*Tg*TH-MID - *Tg*TH-HIGH	-332	-1,988	1,307
Male	WT - *Tg*TH0	1,949	342	3,497
Male	WT - *Tg*TH-MID	290	-1,276	1,824
Male	WT - *Tg*TH-HIGH	-43	-1,472	1,384
Female	Control - *Tg*TH0	-1,001	-2,544	492
Female	Control - *Tg*TH-MID	-2,661	-4,205	-1,175
Female	Control - *Tg*TH-HIGH	-2,993	-4,339	-1,608
Female	WT - Control	2,951	1,708	4,209
Female	*Tg*TH0 - *TgTH*-MID	-1,659	-3,435	88
Female	*Tg*TH0 - *Tg*TH-HIGH	-1,992	-3,650	-342
Female	*Tg*TH-MID - *Tg*TH-HIGH	-332	-1,988	1,307
Female	WT - *Tg*TH0	1,949	342	3,497
Female	WT - *Tg*TH-MID	290	-1,276	1,824
Female	WT - *Tg*TH-HIGH	-43	-1,472	1,384

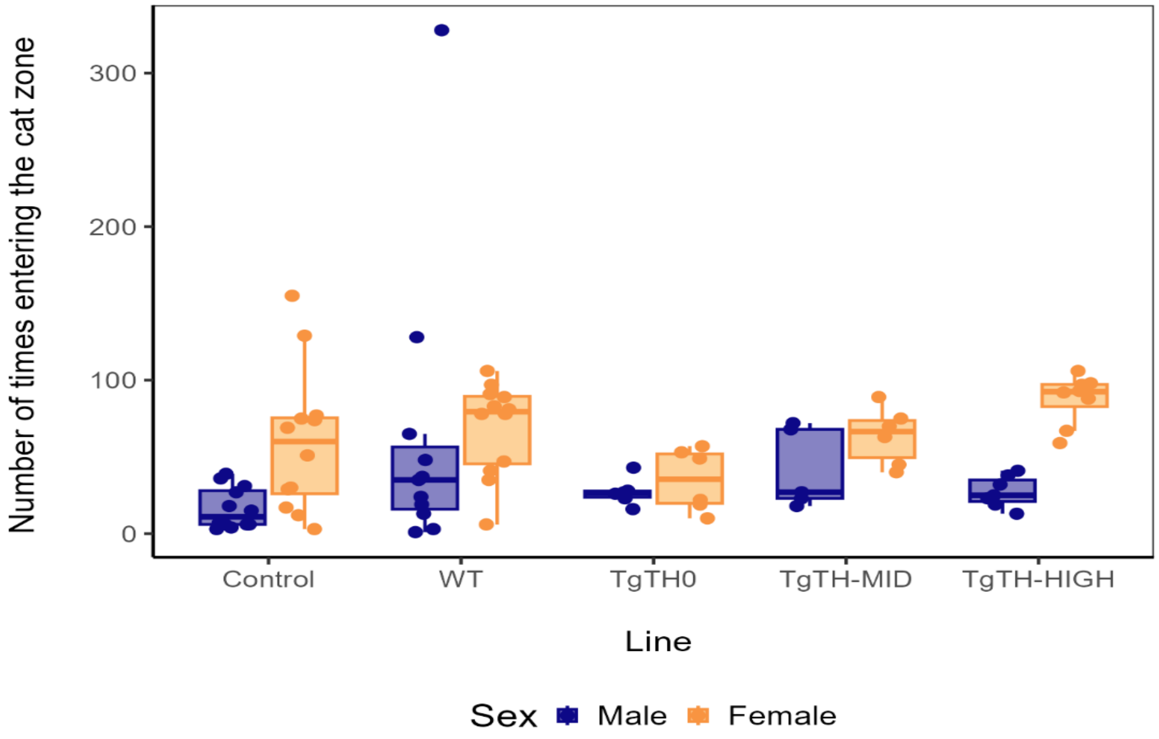

Figure S7. Frequency to enter the cat zone. Distribution of the observations, by line and sex (where males blue and females orange). Number of rats per male/female line group: Control = 12/12, WT = 11/12, *Tg*TH0 = 6/6, *Tg*TH-MID = 5/6 and *Tg*TH-HIGH = 7/8. Source data are provided as a Source Data file.

Table S11. Frequency of entering the cat zone. Posterior means and 95% credible intervals (CrIs; see Figure 5).

Sex	Mean	95% CrI lower limit	95% CrI upper limit
Male	28.766	22.989	35.911
Female	65.530	52.671	81.493

Table S12. Frequency of entering the cat zone. Posterior mean difference between lines and 95% credible interval (CrI; see Figure 5). Differences whose 95% credible interval does not include the value of zero are considered relevant and are highlighted in grey.

Sex	Mean	95% CrI lower limit	95% CrI upper limit
Female -Male	36.764	21.879	54.261

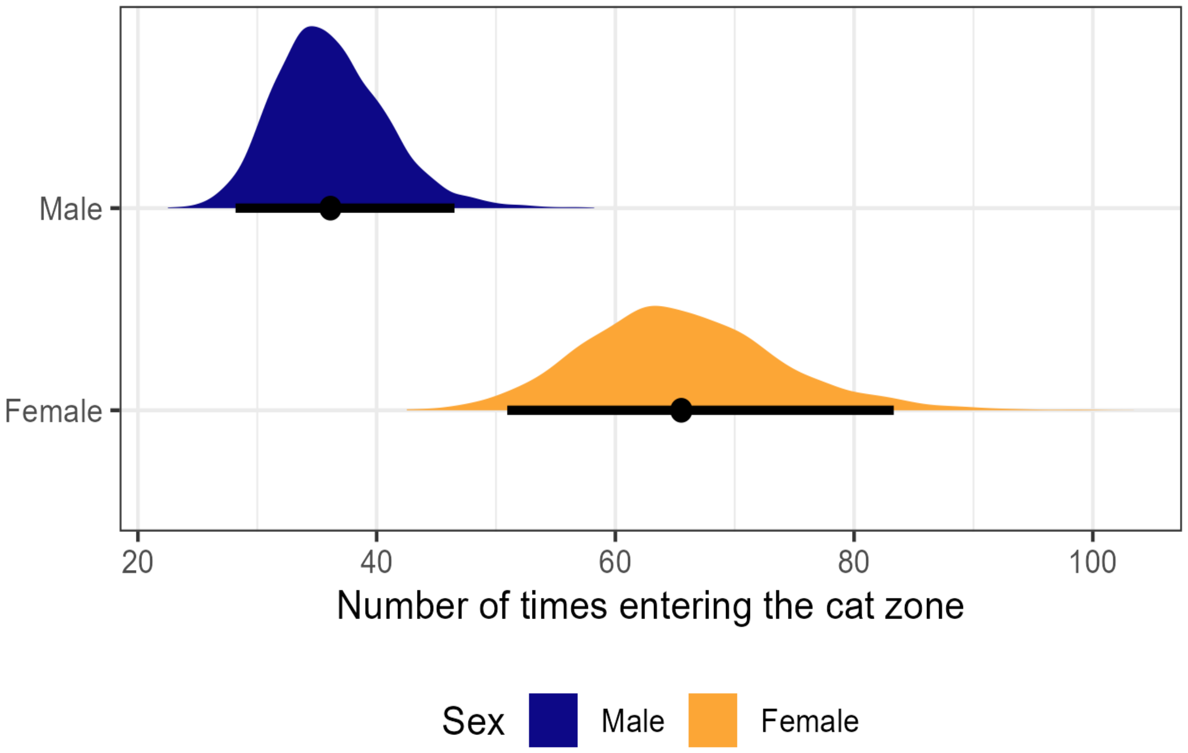

Figure S8. Frequency to enter the cat zone (including the extreme value). Posterior distribution of the means and 95% credible intervals, by sex (where males blue and females orange). Number of rats per sex: Male = 41 and Female = 44. Source data are provided as a Source Data file.

References

1 Mead, R. *The Design of Experiments: Statistical Principles for Practical Application*. 620 (Cambridge University Press, 1990).

2 Webster, J. P., Lamberton, P. H. L., Donnelly, C. A. & Torrey, E. F. Parasites as causative agents of human affective disorders?: The impact of anti-psychotic and anti-protozoan medication on *Toxoplasma gondii's* ability to alter host behaviour. *Proceedings of the Royal Society (London), Series B.* **273**, 1023-1030 (2006).
